# The Apoptosis Paradox in Cancer

**DOI:** 10.3390/ijms23031328

**Published:** 2022-01-25

**Authors:** Ornella Morana, Will Wood, Christopher D. Gregory

**Affiliations:** University of Edinburgh Centre for Inflammation Research, Queen’s Medical Research Institute, 47, Little France Crescent, Edinburgh BioQuarter, Edinburgh EH16 4TJ, UK; omorana@ed.ac.uk (O.M.); w.wood@ed.ac.uk (W.W.)

**Keywords:** apoptosis, cell death, tumour, cancer, cancer therapy, immune system, repair, regeneration, macrophage, extracellular vesicle

## Abstract

Cancer growth represents a dysregulated imbalance between cell gain and cell loss, where the rate of proliferating mutant tumour cells exceeds the rate of those that die. Apoptosis, the most renowned form of programmed cell death, operates as a key physiological mechanism that limits cell population expansion, either to maintain tissue homeostasis or to remove potentially harmful cells, such as those that have sustained DNA damage. Paradoxically, high-grade cancers are generally associated with high constitutive levels of apoptosis. In cancer, cell-autonomous apoptosis constitutes a common tumour suppressor mechanism, a property which is exploited in cancer therapy. By contrast, limited apoptosis in the tumour-cell population also has the potential to promote cell survival and resistance to therapy by conditioning the tumour microenvironment (TME)—including phagocytes and viable tumour cells—and engendering pro-oncogenic effects. Notably, the constitutive apoptosis-mediated activation of cells of the innate immune system can help orchestrate a pro-oncogenic TME and may also effect evasion of cancer treatment. Here, we present an overview of the implications of cell death programmes in tumour biology, with particular focus on apoptosis as a process with “double-edged” consequences: on the one hand, being tumour suppressive through deletion of malignant or pre-malignant cells, while, on the other, being tumour progressive through stimulation of reparatory and regenerative responses in the TME.

## 1. Introduction: The Dual Role of Apoptosis in Cancer

**Figure 1:**
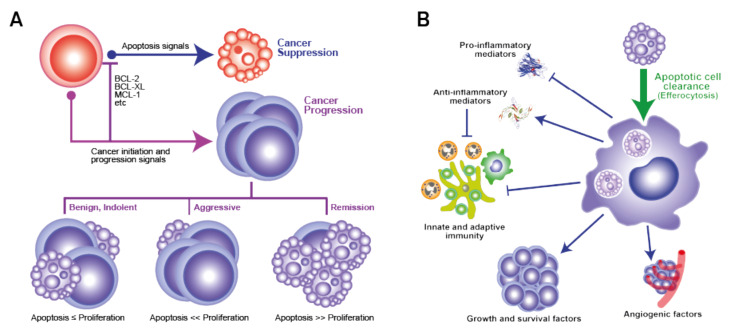
The ‘apoptosis paradox’ in malignant disease (**A**): Apoptosis as a cell-autonomous process is tumour-suppressive (top, blue arrow). Protection from apoptosis (e.g., by anti-apoptotic BCL-2 family members) permits survival of mutant cells and outgrowth of premalignant or malignant populations. Fractional apoptosis within these populations, dependent upon the relative levels of proliferating and dying cells, yields different growth rates. Therapeutic efficacy is achieved when cell death exceeds cell birth (remission, bottom right). (**B**): The best-known functional programmes of phagocytes (especially macrophages) engaging in efferocytosis. All have pro-oncogenic features. Note that immune responses (shown here as suppressed) may also be stimulated by apoptotic cells in certain contexts.

Apoptosis represents a tightly regulated and evolutionarily conserved cell death programme, performing key functions in normal physiological processes such as embryogenesis and adult tissue homeostasis, but also well renowned for its role as a tumour suppressor mechanism. Apoptosis is the normal physiological cell death response to many stimuli, infection or damage, including that which follows cytotoxic drug treatments or radiotherapy programmes, that generate irreparable DNA lesions. The molecular machinery in apoptosis is well characterised and critically requires the activation of caspase proteases, either through an intrinsic pathway initiated through mitochondrial outer membrane permeabilization (MOMP) or triggered at the cell surface by the activation of death receptors, such as Fas and DR4/5, by their death-inducing ligands, for example, FasL and TRAIL, respectively [1]. The role of apoptosis in cancer has received much attention, with resistance to apoptosis being widely accepted as an acquired characteristic of cancer cells, endowing them with survival advantages that promote tumour evolution and outgrowth, as well as treatment failure [2]. Consequently, the efficacy of cancer treatments is strictly dependent not only on the cellular damage they cause, but also on the cells’ ability to activate their apoptosis programme. Over recent decades, research in cancer therapy has largely focused on the development of improved drugs and radiation therapies aimed at inducing maximal tumour cell death with resultant regression of tumour volume and blockade of aggressiveness. Accordingly, significant efforts have been made to inhibit the underlying molecular mechanisms by which tumour cells may evade apoptosis. In recent years, however, more attention has been given to observations that, in diverse cases, high-grade cancers with poor prognosis often contain relatively high levels of constitutively apoptotic cells (Table 1); we and others have proposed that dying tumour cells may help determine net survival and expansion and evolution of the tumour population as a whole (Figure 1).

The paradox of pro- versus anti-tumour effects of apoptosis raises important questions. For example, is the cell death that is induced by anti-cancer therapeutics a wholly advantageous approach? Could therapy-induced cell death stimulate opposite effects by encouraging repair and regeneration responses that result in failure of therapy, or promotion of aggressive clonal evolution and relapse? The controversial concept of life and death disharmony in cancer is giving rise to new research perspectives. For example, new needs are emerging in the area of cancer therapy, including the understanding of the molecular mechanisms underlying apoptotic cell death responses by the identification of novel targets in viable neighbours in the TME. Here, we aim to clarify some essential concepts regarding the cellular birth-death imbalance that occurs in tumours, in order to help improve understanding of the opposing effects caused by apoptotic tumour cell death in the suppression versus progression of cancer. Rather than providing an exhaustive and lengthy review of the literature, it is our intention to restrict our discussion to areas which, in our view, represent important, under-investigated aspects of the apoptosis-conditioned TME.

## 2. Programmed Cell Death in Aggressive Tumour-Cell Populations

It is well established that cell loss through substantial cell death occurs constitutively in malignant disease. Where cell loss has been quantified, observed tumour volume doubling times are actually far slower than the potential tumour doubling times that are simply based on the proliferation rates of the constituent tumour cells [28]. This is true of diverse, aggressive cancer types such as colorectal carcinoma, melanoma and non-Hodgkin lymphoma (NHL). While necrotic tracts of uncontrolled cell death are common in aggressive cancers, so too is the best-known form of programmed cell death, apoptosis. Even in very rapidly growing cancers, cell death can be substantial: for example, in Burkitt’s lymphoma (BL) a B-cell NHL and one of the fastest growing of all human cancers, the rate of cell loss has been calculated to be about 70% of the rate of cell gain [29]; apoptosis is substantial, generating the characteristic starry-sky histological picture of BL [22]. Furthermore, it has been estimated that around 70% of the cells in aggressive glioblastoma are apoptotic [17]. Therefore, although evasion of apoptosis is an undoubted oncogenic mechanism, tumour cell populations in a wide variety of malignancies are unable to inherently and continuously suppress the apoptosis programme in all of their constituent cells. Apoptosis is also an important cellular response to cancer therapy and, indeed, induction of cell death to remove all cancer cells is the aim of the vast majority of non-surgical cancer therapies. For many years, the role of programmed cell death in tumour development has remained focused on suppression being the defining principle. Not until relatively recently did the concept that cell death may promote, as well as suppress, tumour growth come to the fore, even though it has been known since the middle of the last century that lethally-irradiated tumour cells can markedly promote tumour growth in rodent models [30,31]. Here, we will concentrate mainly on the local roles of apoptosis, potential and proven, in the TME.

## 3. Apoptosis and the TME

Following Paget’s seed and soil concept [32], primary and metastatic cancer development relies fundamentally on the tumour ‘niche’, the TME. Evidence shows that molecular adjustment of the extracellular matrix and genetic alterations represent some of the shared main hallmarks of all tumours, gradually accumulating through the stages of tumourigenesis, and becoming increasingly pronounced as the tumour progresses. Further complicating this framework is the heterogeneous cellular composition of the TME, encompassing different cell lineages at diverse levels of differentiation, which may dictate the extent of its role in reparatory activities [33]. Such cells include neoplastic cells (themselves clonally heterogeneous), endothelial cells, infiltrating and/or expanding innate immune cells and a variety of associated tissue cells including fibroblasts, as well as the normal counterparts of the evolving mutant clones. Complex interrelationships between cellular and non-cellular components of the TME orchestrate tumour establishment, progression, invasion and response to therapies.

We have already noted firstly that apoptosis is a common feature of the TME and, secondly, that apoptosis indices have been studied in multiple types of cancer and relatively higher levels of apoptosis are generally associated with more aggressive tumours (Table 1). From a tumour-cell-autonomous perspective, this is surprising, given the tumour-suppressive role of apoptosis in this context. However, from the perspective of the tumour-cell population as a whole—indeed the whole cancerous tissue—fractional apoptosis may prove to be an essential component of the TME because of the range of cellular and molecular responses it can engender (*vide infra*). We may also speculate that these principles could regulate oncogenesis throughout tumour evolution, from pre-malignancy to metastasis.

While causative relationships between the aggressiveness of malignant disease and the levels of tumour-cell apoptosis remain largely theoretical, additional correlative evidence can be drawn from observations that high expression of pro-apoptotic effectors may not correlate with low aggressiveness of disease and vice versa. This is illustrated, for example, by pro-apoptotic Bax expression [34] or caspase activation [35] being associated with aggressive disease and by anti-apoptotic Bcl-2 being linked to less aggressive states [36]. Furthermore, cell death may foster genomic instability and niche creation which, in the context of nascent and progressing tumours, could lead to repopulation by tumour cell clones with more aggressive properties [37]. As we will discuss, accumulating evidence indicates that the apoptosis programme also has the capacity to provide significant pro-oncogenic signals—here from a cell population (rather than single-cell autonomy) perspective—which feed into the cell birth/cell death imbalance that leads to cancer (Figure 1A). We believe that innate regenerative responses to apoptosis are hijacked in cancer in order to promote and retain net tumour growth and progression. Of particular importance in this regard is the well-established knowledge that apoptotic cells attract and polarise macrophages to M2-like reparatory and regenerative activation states with the potential to promote cancer evolution and progression through diverse pathways (Figure 1B).

The observation that rapidly proliferating cancers have more frequent incidence of apoptosis amongst their transformed cells may reflect a parallel that is observed in normal tissues: that high rates of proliferation are often associated with high rates of cell death. As we have discussed elsewhere [38], triggers for constitutive tumour-cell apoptosis in the TME of an aggressively growing lesion are likely to include (a) nutrient and oxygen deficiency as a result of tumour-cell proliferation outpacing angiogenesis (indeed, inhibition of angiogenesis is known to inhibit tumour growth by markedly stimulating apoptosis, thereby creating a dormant state [39]), and (b) clonal ‘cancer hallmark insufficiency’: pre-malignant or malignant clones which have acquired genetic mutations but fail to survive in the TME because they are deficient in acquired characteristics of cancer cells [2]) needed for their survival. In this context, apoptosis of ‘loser cells’ in the TME of carcinomas (epithelial cancers) also arises through cell competition. Additional stimuli that initiate and propagate apoptosis are therapeutic modalities, anti-tumour immune mechanisms and the process of ‘apoptosis-induced apoptosis’ (AiA) [40].

Thus, the TME provides essential signals for the emergence, evolution and expansion of tumour populations through establishment and maintenance of cell birth/cell death ratios at levels > 1 (Figure 1A). During tumour evolution and growth, dynamic adaptation of the TME is required in order to favour this ratio and, as we expound below, emerging evidence indicates that responses to apoptosis in the TME likely provide critical contributory mechanisms.

### 3.1. Cellular and Molecular Responses to Apoptosis in the TME

#### 3.1.1. Phagocytic Clearance and Anti-Inflammatory Signalling

Far from being inert entities, apoptotic cells induce varied responses in their neighbourhood through the active exposure and release of an array of bioactive components (see [38] (especially Table 3.1), [41,42,43,44] for detailed reviews). Indeed, the secretomes of dying cells that are generated during cancer therapy have important functional effects in facilitating tumour evolution and metastasis [45]. The most renowned characteristic of an apoptotic cell is its ability to elicit its own phagocytosis by neighbouring tissue cells of various lineages, including dendritic cells, fibroblasts, myocytes, endothelial cells, mesangial cells, Sertoli cells, Paneth cells and most predominantly macrophages, the most readily observed phagocytes of apoptotic cells and the most extensively studied. Indeed, the process of apoptotic-cell engulfment is so swift that free apoptotic cells are rarely observed in situ. Orchestration of the phagocyte’s responses in mediating clearance of apoptotic cells (a process often referred to as efferocytosis) requires transduction of ‘find-me’ and ‘eat-me’ signals generated by the dying cells (reviewed recently [43]). Examples of the ‘find-me’ signals that attract phagocytes to the locations of apoptosis in mammals include (a) bioactive lipids lysophosphatidylcholine (LPC) [46] and sphingosine-1-phosphate (S1P) [47], (b) the classical chemokine CX_3_CL-1 (fractalkine), which is known to be associated with extracellular vesicles (EVs) released by apoptotic lymphoid cells [48] and (c) nucleotides ATP and UTP, which are released through effector caspase-3-mediated activation of the membrane channel pannexin I [49,50]. The latter controls transmembrane passage of molecules up to approximately 1kDa through the formation of ion channels in the plasma membrane [51]. These different classes of chemoattractants all activate chemotaxis of mononuclear phagocytes by binding to specific G protein-coupled receptors. It is noteworthy that the responses of phagocytes to these factors are not limited to migration alone but can include, in addition, stimulation of engulfment mechanisms (for example, through MFG-E8 induction by fractalkine [52] and polarisation of macrophages from pro-inflammatory to anti-inflammatory states, as well as the promotion of lymphangiogenesis and metastasis in murine tumour models (activation of S1PR1 by S1P [53,54]). Leukocyte chemotactic control by release of soluble factors from apoptotic cells can also select for the accumulation of mononuclear phagocytes by inhibiting the migration of granulocytes. This is seen in the case of lactoferrin (LTF) which is released during apoptosis and restricts the migration of neutrophils and eosinophils [55,56]. Although this may represent an important aspect of the anti-inflammatory effect of apoptosis, its role in determining the cellularity of the TME has not been investigated.

The most renowned ‘eat-me’ signal is the anionic phospholipid, phosphatidylserine (PS), which is actively externalized in the plasma membrane leaflet during apoptosis. This is achieved through the coordinated caspase-dependent (a) inhibition of flippase activity (P4-ATPase ATP11C), which otherwise maintains PS localisation in viable cells to the inner aspect of the plasma membrane leaflet, and (b) activation of scramblase activity (Xkr family member Xkr8), which accelerates the redistribution of PS across the membrane bilayer [57]. While PS exposure may be necessary for an apoptotic corpse to be engulfed, it is however, insufficient. Additional, ill-defined, components are required, such as carbohydrate changes and binding sites for thrombospondin or other bridging molecules, as well as loss of ‘don’t-eat-me’ signals, such as CD47 and CD31 [38]. Multiple phagocyte receptors that participate in efferocytosis can either directly (BAI1, TIM4, Stab2, TLT and RAGE) or indirectly (MERTK, α_v_β_3/5_, LRP1) engage with PS exposed on apoptotic cells. Indirect interaction with PS is achieved via PS-binding bridging receptors such as Gas6 in the case of MERTK, MFG-E8 for the integrins α_v_β_3_ and α_v_β_5_, while C1q bridges to LRP1. Additional receptors, such as the scavenger receptors CD36 and SR-A, as well as the PRRs CD14 and MBL, recognise apoptotic cells via PS-independent mechanisms (reviewed previously [38,41,43,44]).

Similar to the death effector machinery of apoptosis, the phagocytosis programme in apoptotic corpse clearance is conserved from worms to mammals, engulfment involving two pathways, originally identified in the nematode worm, *Caenorhabditis elegans*. These have been reviewed elsewhere ([41,43,58]), each pathway comprising three genes: (a) CED-1, (cell death defective-1) -6 and -7 and (b) CED-2, -5 and -12. Mammalian orthologues of CED-1 are MEGF-10 and LRP1, CED-6 is the engulfment adapter GULP1; CED-7 is represented by ABCA1/7. In the second pathway, CED-2, -5 and -12 are represented in mammals by CrkII, Dock180 and ELMO, the latter two proteins acting as an unconventional two-component guanine nucleotide exchange factor for activating the Rho-family GTPase, Rac-1 (CED-10 in *C. elegans*). Either pathway can activate Rac-1 (although the connectivity been GULP1 and Rac activation remains unclear) and this leads to cytoskeletal rearrangement, actin polymerisation and engulfment with subsequent phagosome maturation and lysosomal degradation of phagocytosed cargoes (reviewed recently [43]). Engulfment of apoptotic cells is typically accompanied by activation of anti-inflammatory responses involving up-regulation of multiple factors, including TGF-β1, IL-10, PGE_2_, PGI_2_ and PAF. In concert, key pro-inflammatory mediators, such as TNFα, IL-1β, IL-8 and IL-12, are downregulated. Details of the molecular mechanisms linking recognition to phagocytosis and inflammation control are emerging. Some receptors, CD14, for example, are mainly involved in tethering of apoptotic cells to phagocytes, whereas others, for example, BAI1 and Stab2, clearly signal Rac-dependent phagocytosis and downstream lysosomal processing. Modification of the aforementioned canonical phagocytosis signals by a process known as LAP (LC3-associated phagocytosis) can speed up lysosomal fusion and degradation and promote anti-inflammatory cytokine production, while abrogation of LAP slows fusion and degradation and promotes pro-inflammatory cytokine release. The details of LAP signaling, including its initiation, are not fully understood, but involve the formation, following receptor-mediated phagocytosis, of a phosphatidylinositol 3-kinase (PI3K) complex comprising at least five proteins, UVRAG, Rubicon, Beclin 1, VPS15 and VPS34. Activation of this PI3K complex results in ligation of LC3 to the lipid membrane of the phagosome and consequent acceleration of lysosomal fusion, cargo degradation and immunosuppressive signaling [43]. It has also been established recently that chloride sensing and flux involving the solute carrier (SLC) proteins SLC12A2 and SLC12A4 play important roles in triggering anti-inflammatory efferocytosis [59].

Tumour-associated macrophages (TAMs) constitute a significant proportion of the cellular compartment of the TME in diverse malignancies where, in many cases, they are clearly active in clearance of apoptotic cells. As we discuss later, TAM accumulation and pro-oncogenic activation, at least in certain cancers, is closely coupled to tumour growth and angiogenesis. However, little is yet known of the relative contributions of the various find-me, eat-me and anti-inflammatory signaling mechanisms and molecules described above in the pro-oncogenic responses of TAMs to apoptotic tumour cells. Notably, the protein tyrosine kinase MERTK (an indirect PS receptor) is functional in signaling not only phagocytosis of apoptotic cells but also anti-inflammatory/immunosuppressive responses; its inhibition can suppress cancer growth (reviewed in [60]). It seems likely that phagocyte receptors will orchestrate context-dependent immune responses to apoptotic tumour cells whether dependent on, or independently of, PS; future work will define what receptors are important in specific tumours. An informative example is post-partum breast carcinoma, which tends to present as an aggressive, metastatic disease. In a mouse model that used the involuting mammary fat pad as a breast cancer transplant microenvironment, the critical importance of constitutive apoptosis, MERTK-dependent efferocytosis and TGFβ production were demonstrated [7].

#### 3.1.2. Other Cells, Other Signals, Other Responses

A poorly understood aspect of the apoptosis programme, not least in the context of the TME, is the apoptotic cell’s role in shaping its tissue environment beyond its own quiet clearance. It has long been known that apoptotic cells can directly influence non-phagocytic responses in neighbouring cells that include cell-fate decision making—survival, proliferation, differentiation and death—in addition to the better known migratory, inflammatory and immunomodulatory responses we have described above. Evidence suggests that all mammalian cell types, whether phagocytic or not, may have inherent capacity to react to apoptosis of neighbouring cells via a MAPK-centric mechanism [61]. Recent work has demonstrated that apoptotic stress in cells can activate survival responses in other cells within the population, for example, via FGF-induced transient upregulation of the apoptosis suppressor, Bcl-2 [62]. These principles of pleiotropic responses to apoptosis by multiple different cell types in the TME, whether phagocytic or not, and including healthy tumour cells, immune cells, fibroblasts, endothelial cells and others, form the basis of a complex network of programmes that help determine whether a tumour is benign, malignant, regressing or relapsing.

Mitogenic responses to apoptosis were first described in *Drosophila* during development and in responses to wounding. So-called ‘compensatory proliferation’ or apoptosis-induced proliferation (AiP) appears either to be dependent on, or independent of, complete apoptosis induction (i.e., without resultant cell death) with significant evidence pointing to cell death-independent properties of the apoptosis machinery, notably, caspases. There is little doubt, however, that AiP is conserved from hydra to mammals and that apoptosis can drive regenerative responses through WNT, Hh, TGFβ/BMP, P53, JNK and MAPK/CREB signaling [38,44,63]. Intriguingly, p38-Mapk-triggered Wnt3 release by apoptotic cells that governs head regeneration in hydra [64,65] is also responsible for driving proliferation of murine hair follicle stem cells [66]. That mammalian apoptotic cells are functionally active in secreting bioactive molecules while their membranes remain intact and, independently of their uptake by phagocytes, has been elegantly reported recently. Thus, apoptotic leukocytes were found to release specific arrays of metabolites through maintenance of certain metabolic pathways and through metabolite release mediated by caspase-activated pannexin I channel opening. Common metabolites produced by lymphocytes and macrophages responding to different apoptotic stimuli included AMP, ATP, GMP, creatine, spermidine and glycerol-3-phosphate. Significantly, the apoptosis-induced ‘metabolite secretome’ was able to activate proliferative and reparatory responses, as well as anti-inflammatory signaling in neighbouring healthy cells [67]. The exploitation by pathogenic bacteria of metabolites released by apoptotic gut epithelial cells [68] provides further evidence that apoptosis generates a metabolite landscape that has important implications for the TME.

Below, we will consider in detail how apoptosis in the TME can help drive the proliferative, migratory and differentiation processes required for angiogenesis, an essential characteristic of malignant disease. A further example of apoptosis promoting differentiation is the induction of myogenesis by the interaction of PS exposed on apoptotic myoblasts with the PS-receptor BAI1 on their healthy counterparts [69]. Cell fusion is thought to contribute to several aspects of oncogenesis, such as aneuploidy and motility; it is tempting to speculate that apoptotic cells in the TME may contribute to fusogenic tumour-promoting activities. To our knowledge, this has not been investigated to date.

Finally, as we have already intimated in Section 3 above, the expansion of apoptosis signalling within tumour-cell populations by AiA may also have double-edged implications for tumour biology. AiA is known to delete cohorts of cells during mouse hair cycling synchronously via the production of TNFα by apoptotic cells, which, in turn, propagates in healthy neighbours. The same study showed this to be evolutionarily conserved with the same signalling mechanism driving AiA in the fly wing imaginal disc [40]. As well as its potential for promoting therapeutic efficacy through bystander tumour-cell death induction and for removing pre-malignant clones to suppress oncogenicity, the opposite may again prove to contribute to tumour outgrowth: AiA has the promise to drive tumour-promoting responses in the TME through any or all of the mechanisms we have discussed.

### 3.2. A Cell Death-Driven TME Feature: The Onco-Regenerative Niche

**Figure 2:**
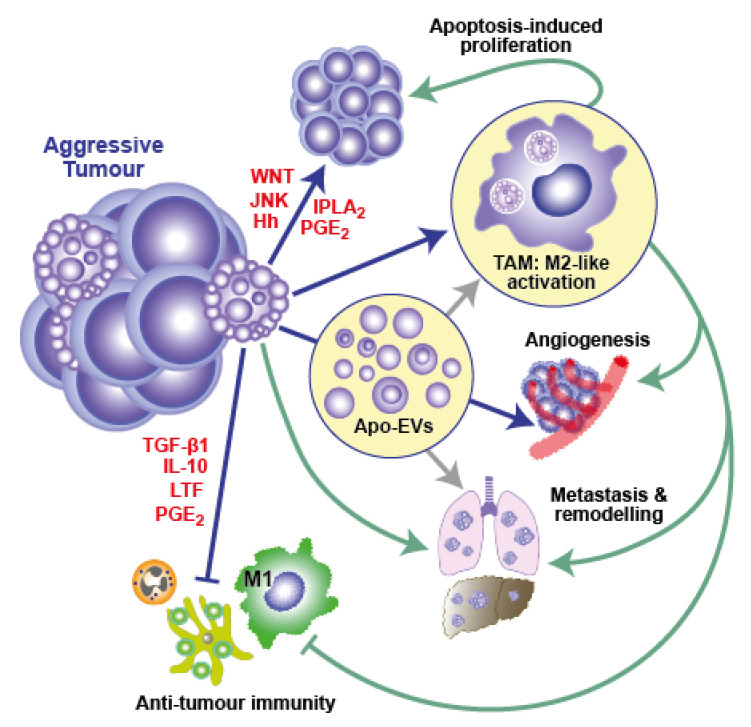
Pro-oncogenic effects of apoptosis and the concept of the *Onco-Regenerative Niche* (ORN). Apoptotic tumour cells have broad effects on the tumour population as a whole and provide the driving force in a conceptual sub-sector of the tumour micro-environment, which we have termed the ORN. Solid blue arrows and associated molecules highlight proven pathways that we and others have elucidated in recent years. Green lines indicate putative pathways in the ORN that we have identified through in situ transcriptomics of TAMs. Grey arrows indicate potential functional pathways of Apo-EVs. See text for further details.

In line with cancers displaying attributes of wounds that fail to heal or, perhaps more accurately, wounds that continue to repair, much evidence that has emerged over the last decade would suggest apoptosis-driven tissue repair and regenerative responses are likely to play important roles in generating and supporting the TME [6,22,38,42,70,71,72,73] (Figure 2). We have termed this cell death-driven aspect of the TME, the “onco-regenerative niche” (ORN) [38,42,74]. The ORN was coined in order to provide a conceptual framework around the characteristics and consequences of cell death in the TME, given the fundamental axiom—as we have discussed—that dying cells are far from inert entities. It encompasses the intercellular communication modalities and the cell and tissue programmes that are activated as a consequence of apoptosis in the TME to promote oncogenesis and malignant disease progression through apoptosis-driven regenerative and tissue repair mechanisms that promote tumour expansion and invasiveness, while suppressing anti-tumour immunity (Figure 2). Sources of both apoptotic and apoptosis-responding cells may include transformed cells, as well as other cellular constituents, of the TME; communicating mechanisms comprise intercellular contact, soluble factors and EVs. Although potentially broad in therapeutic application, since many different types of cancer display high rates of apoptosis, little is yet known about the key operational mechanisms of the ORN. Here we highlight TAMs as cellular players and apoptotic tumour cell-derived EVs (Apo-EVs) as intercellular communication vehicles, proposing roles for their involvement in immunosuppression and for angiogenesis in the TME.

#### 3.2.1. Potential Key Elements of the ORN: TAMs and Apo-EVs

##### TAMs

Macrophages are major cellular components of multiple classes of tumours and, although they have proven power to kill tumour cells in their classically activated state (often known as M1), their predominant function in cancer tends to be to support tumour growth via multiple mechanisms, including activation of angiogenesis, production of growth and survival factors, and support of invasion and metastasis, while also suppressing anti-tumour immunity. This reparatory macrophage phenotype—often referred to as M2-like—is typical of macrophages responding to apoptotic cells (Figure 1B and Figure 2). Similar to apoptosis, TAM accumulation is correlated with poor prognosis in diverse cancer types. It has been proposed that the TME conditions the activation state of TAMs, inducing a switch from anti- (M1) to pro-tumour (M2-like) [53,75,76,77,78]. Of note, apoptotic cells are not only key players in inducing M2-like activation of macrophages, but are also readily engulfed by M1 macrophages and dominantly suppress M1 anti-tumour activity [79,80]. That apoptosis profoundly and dominantly affects macrophages is further demonstrated by its ability to imprint a reparatory memory on macrophages and elicit a dominant migratory response signal in flies [81,82], observations which suggest that apoptosis may provide dominant signals to TAMs in the TME.

As we have discussed in detail above, TAMs are the most commonly encountered cells that, identified through their obvious efferocytotic activity, clearly interact with apoptotic cells in the TME. While it seems obvious that efferocytosis could provide TAMs with mechanisms for nutrient recycling and transmission from apoptotic to healthy tumour cells, little is yet known about the biology of this process or its relevance to tumour growth. It is very clear, however, that efferocytosis functionally programmes macrophages to produce arrays of cytokines and other bioactive molecules with pleiotropic effects that can promote tumour growth through proliferation signalling, regeneration and repair responses and anti-tumour immune silencing (Figure 1B and Figure 2). Amongst the most renowned of these are the anti-inflammatory mediators TGF-β1, IL-10 and PGE_2_, all of which are known to have pleiotropic effects on their target cells. A case in point in relation to the last of these factors is the ‘phoenix rising’ repopulation pathway, which was first described as an apoptotic cell-driven regeneration pathway active in skin wound-healing and liver regeneration [70]. The authors defined the pathway based on the activation of effector caspase-3 and -7, which generate arachidonic acid through iPLA_2_ cleavage and activation. Subsequent conversion of arachidonic acid by cyclooxygenases 1 and 2 into PGH_2_ is followed by conversion into PGE_2_ by PGE_2_ synthase. PGE_2_ was found to be the critical effector of stem cell proliferation, repair and regeneration [70]. While this pathway may generate PGE_2_ release from apoptotic cells themselves, it also has elements of an efferocytic response to apoptosis, since cyclooxygenases are activated in macrophages by apoptotic cells and PGE_2_ is a common resultant product [83]. The caspase-3-dependent production of PGE_2_ has also been reported to support the stimulation of tumour re-growth in mice following therapy-induced apoptosis in models of breast cancer, melanoma, bladder carcinoma and glioblastoma [4,6,16,84]. PGE_2_ has additional oncogenic roles in suppressing adaptive immunity and promoting angiogenesis [53,83].

Because of the inherent plasticity of macrophages, the polarisation of TAMs is finely tuned by their environmental cues, including apoptotic cells. In order to understand the activation status of TAMs interacting with apoptotic tumour cells in situ, we previously investigated the transcriptomic profile of efferocytosing TAMs in starry-sky NHL (SS-TAMs) isolated from their TME by laser capture microdissection. SS-TAMs were found to display an M2-like status, characterised by the upregulation of gene clusters known to be associated with (1) apoptotic cell clearance and anti-inflammatory responses (e.g., *MSR1*, *LRP1*, *MERTK*, *AXL*, *GAS6*, *CD36*, *CD93*, *LGALS3*, *ABCA1* and *TGFB1*), and (2) survival, proliferation, angiogenesis, repair and remodeling (e.g., *MRC1*, *ANPEP*, *GPNMB*, *HMOX1*, *PLAU*, *CTSB*, *CTSD*, *CTSL*, *IGF1*, *PDGFC*, *FN1* and *MMP12*) [22]. Although the relative importance of most of these genes remains unproven with respect to their requirements for NHL growth, we have shown, subsequently, that NHL TME deficiencies in *MERTK* or *LGALS3* (the gene encoding galectin-3) severely impair starry-sky NHL growth [80,85]. Together, these studies demonstrate in a tumour context that efferocytosis imparts oncogenic properties to the TME. Much remains to be learned about how apoptosis drives the M2-like phenotype of TAMs in the ORN, though it is notable that TAM production of lactate appears to be an important pathway in metabolic programming of pro-tumour, M2-like macrophage activation [86]. Intriguingly, efferocytosis stimulates aerobic glycolysis and lactate production in phagocytes leading to the generation of an anti-inflammatory milieu via SLC family activation [87].

##### Apo-EVs

While most of what we know about the molecular mechanisms underlying the influence of apoptosis on the TME relates to the responses of phagocytes to apoptotic cells or ‘corpses’, relatively little is yet known about the responses of phagocytes and other viable cells to Apo-EVs that are actively produced during apoptosis. EVs are membrane-delimited subcellular particles that are released from both healthy and apoptotic cells. In simplified terms, on the basis of physical properties and modes of biogenesis/release, EVs from healthy cells are generally subdivided into (1) exosomes, which are in the range ~30–100 nm in diameter and derived from the endosomal, multivesicular body system, and (2) microvesicles, ~50–1000 nm and derived by budding from the plasma membrane. Apo-EVs are known by numerous terms including ‘apoptotic blebs’, ‘apoptotic vesicles’, ‘apoptotic microparticles’ and, commonly, ‘apoptotic bodies’. We support the terminology, adopted by Poon and colleagues, that, from the perspective of vesicle size, Apo-EVs are categorized as being <1000 nm, while apoptotic bodies are regarded as being 1000 nm and above (typically 1000–5000 nm) [88]. Since apoptosis is well defined as a cellular programme in which EV production is markedly increased, albeit through ill-defined mechanisms, there seems little doubt that Apo-EVs will be in plentiful supply as intercellular communication vehicles in the ORN. As with EVs from healthy cells, Apo-EVs carry diverse biological cargoes, including DNA, RNA, wide arrays of proteins, lipids, metabolites and small molecules together with, at the larger end of the scale, organelles such as ribosomes and mitochondria [74,88,89,90,91]. 

Apo-EVs can activate find-me and eat-me responses of phagocytes, for example through fractalkine and PS exposure, respectively, and can modulate inflammatory and adaptive immune responses, for example, through transfer of cytokines and antigen presentation, either directly to T cells or via engulfment by dendritic cells [90]. As with all EV populations, those released from apoptotic cells are heterogeneous; one of the major challenges in deciphering the biology of Apo-EVs—those released specifically as a consequence of the apoptosis programme—is discriminating the Apo-EVs from those ‘background’ EVs that are produced, in the same temporal frame, independently of apoptosis. We refer the reader to recent reviews on Apo-EVs, their cargoes and functions [74,88,89,90]. Of particular relevance to the biology of the ORN is the recent observation that dying glioblastoma cells can transfer splicing factors to their healthy neighbours, endowing them with more aggressive characteristics [17]. Detailed guiding principles as to the functional attributes of Apo-EVs in the TME await definition and much remains to be learned about the target cell preferences (if any) of Apo-EVs in the ORN and their modes of entry into target cells, as well as the intracellular destinations and functional capacities of their cargoes. 

#### 3.2.2. Apoptosis-Induced Angiogenesis: A Key Tissue Programme of the ORN?

Without an angiogenic programme, malignant tissues are unable to expand. Rapidly growing, aggressive cancers can outgrow their essential vasculature and commonly generate regions of severe hypoxia and anoxia that are incompatible with cell viability. Inhibition of angiogenesis has long been known to promote apoptosis markedly and, thereby, to inhibit cancer growth [39,92]. However, apoptotic cells have the capacity to drive angiogenesis, both through direct effects on vascular progenitor cells and indirectly via macrophage activation [22,93]. Hence, it seems conceivable that apoptosis and angiogenesis are closely coupled in the ORN to provide a dynamic feedback loop that supports cancer growth. This tissue programme, which exemplifies critical intercellular signaling, networks in the TME that are dependent upon apoptosis, remains speculative; however, based on current knowledge of known pathways, it is likely to involve the following principles and players in addition to the essential element of apoptosis signaling occurring in a proportion of the tumour-cell population: (1) accumulation of TAMs through apoptotic cell-derived ‘find-me’ signals; (2) pro-angiogenic activation of TAMs by apoptotic cell-derived factors via soluble mediators, direct intercellular contact and through the actions of Apo-EVs; (3) activation of endothelial cells and their progenitors through soluble factors, intercellular contact and EVs from apoptotic tumour cells and from apoptosis-activated TAMs. Therefore, co-ordinated conditioning of the TME by these mechanisms may be initiated by tumour-cell apoptosis consequent to hypoxia caused by rapid proliferation. This, in turn, feeds positively into the loop wherein further proliferation generates further hypoxia, apoptosis and angiogenesis and the cycle continues. In support of this model, starry-sky NHL TAMs that interact with apoptotic lymphoma cells in situ have a pro-angiogenic, M2-like activation state and are found preferentially in areas coincident with CD31-positive endothelial cells [22]. Furthermore, lymphoma cell-derived Apo-EVs have pro-angiogenic properties (our unpublished observations). Much experimental work is required to determine the extent to which this angiogenesis cycle operates in different cancer types in which apoptosis drives oncogenesis and to define underlying molecular mechanisms. In addition, understanding the angiogenesis mechanisms that follow cell death-inducing therapy and facilitate relapse is an important area of future research. Notably, apoptosis-driven angiogenesis has been reported in a mouse model of relapsing glioblastoma following radiation therapy and appears to require caspase-3-dependent PGE_2_ and VEGF-A production [16].

## 4. Distinct Cell Death Programmes

Regulated cell death programmes represent conserved intrinsic mechanisms evolved to maintain tissue homeostasis [1,94,95,96]. Notably, cells undergoing irreparable genomic mutations activate apoptosis to prevent passage of mutations to progeny, an important tumour-suppressive strategy [97,98,99]. While apoptosis remains the most widely understood cell death programme, additional programmed cell death mechanisms, such as necroptosis, pyroptosis, and ferroptosis, have been classified by the Nomenclature Committee on Cell Death in 2018, amongst more than 20 different cell death modes [94]. Similar to apoptosis, these programmes appear to act—although via different mechanisms and with different sequelae—either as defence/deletion mechanisms occurring in normal tissues or in tumour prevention and eradication. We refer the reader to recent reviews encompassing non-apoptotic cell death programmes, including their perceived roles in cancer [44,96]. Entosis and phagocytosis constitute non-cell-autonomous cell death programmes that follow engulfment of living cells. The latter is mediated via lysosomal degradation of living cells that are phagocytosed following inhibition of ‘don’t-eat-me’ signalling axes, notably CD47/SIRPα and, although little is known of its role in ‘constitutive’ cell death in the TME, antibody-mediated inhibition has proven therapeutic efficacy [100]. We consider the special case of entosis further in Section 4.2 below. 

### 4.1. Apoptosis-Autophagy Crosstalk in Cell Death and Survival in Cancer

The autophagy programme emerged in unicellular organisms as a homeostatic intracellular pathway delivering cytoplasmic material, damaged organelles and misfolded proteins to lysosomes for degradation, prior to apoptosis that appeared only later in metazoans [101]. In the context of cancer, poorly vascularized tumour cells can endure nutrient limitation and hypoxia or other stress conditions, including cancer treatments, by activating autophagic pro-survival mechanisms [102]. Thus, its upregulation can promote aggressiveness and reinforce resistance to cancer therapy [103,104,105]. However, as described for apoptosis, in response to certain stimuli, and also for specific cell/tissue types and tumour grades, autophagy can instead trigger tumour suppression, by initiating a tightly regulated pathway that leads to cell death [106,107]. In this sense, the cell death response acts as a backup strategy for maintaining tissue homeostasis [108]. Consequently, just as with apoptosis, autophagy may exert dual roles in cancer, either encouraging tumour endurance or acting as a self-sacrifice process, aimed at preserving tissue integrity [109,110,111].

Apoptosis and autophagy share key metabolic regulators, suggesting that both pathways are employed to respond to similar cell death/survival stresses. Among others, supported roles have emerged for reactive oxygen species (ROS), as by-products of redox homeostasis, triggering either autophagy or apoptosis in cancer cells, in a dose-dependent manner [112,113]. At early stages of tumorigenesis, autophagy occurs as a quality control mechanism by preserving tissues from damage and injuries, removing damaged organelles and defective proteins and preventing tumour initiation and genetic accumulation, whilst apoptosis is meanwhile suppressed [114]. Conversely, during tumour development, autophagy can instead repair damaged DNA and organelles, thus sustaining tumour expansion and survival. At late stages, when the response to stress becomes too severe, the autophagy programme may even shut down in favour of the apoptosis programme, thereby preventing propagation of the metabolic consequences of mutational stress [115].

Similar to apoptosis, the tumour-promoting role of autophagy is accomplished through shaping the TME and has a strong impact on cancer therapy [116]. The effects of autophagy on the TME-associated immune system elements during oncogenesis are notable and include the accumulation and M2-like polarization of TAMs to a tumour-promoting activation state [117,118,119]. Recent advances have also highlighted autophagy being triggered by EVs, as well as EV release being triggered by autophagy, both these areas being worthy of future investigation to further understand apoptosis/autophagy biochemical crosstalk in cancer [120,121].

### 4.2. Mixtures of Cell Death Processes in the TME

Although the main emphasis of this review is apoptosis in the TME, multiple regulated cell death programmes may, in a similar vein, induce a spectrum of related repair and regenerative responses that have oncogenic potential in different cancer types, in different areas of the same malignant lesion or even juxtaposed alongside apoptosis. Alternative regulated cell death programmes in the TME can differentially determine the outgrowth of different types of liver cancer: hepatocellular carcinoma has been reported to be driven by apoptosis-rich TME, whereas necroptosis favours cholangiocarcinoma [122]. Even unregulated necrosis engenders tissue repair [123] and has capacity both to activate anti-inflammatory responses in macrophages, notably as a consequence of membrane PS exposure [124] and to suppress anti-tumour T-cell responses [125], complementing the view that the proinflammatory effects of necrosis may be critically important for certain types and stages of tumour growth [126]. Necrosis has also been reported to drive tumour repopulation via an HMGB1-dependent pathway after radiotherapy [127]. 

Crosstalk between cell death processes, especially the regulated programmes of apoptosis, autophagic cell death, necroptosis and pyroptosis is known [94]; however, its roles in cancer progression versus suppression remain unclear. What are the consequences for cancer pathogenesis of multiple cell death programmes emanating from common signaling sources? Competition for nutrients is a key component in cancer growth; glucose deprivation can induce apoptosis, necrosis and entosis [128]. The latter regulated non-cell-autonomous cell death programme is characterised by the engulfment and autophagic degradation of cells by their neighbours [129,130]. As with apoptosis, entosis is commonly seen in cancer and can promote competitive evolution of oncogenic lesions through engulfment, degradation and recycling of relatively weaker (“loser”) cells by relatively stronger neighbours (“winners”) [131]. Recent evidence indicates that the apoptosis-inducing ligand TRAIL is capable of eliciting signals for cell death in colorectal cancer via both apoptosis and entosis. Both processes are dependent upon caspase-8, the canonocal apoptotic death-receptor pathway-initiating caspase, although, intriguingly, the entosis effect does not require catalytic processing of caspase-8 as occurs in apoptosis, downstream of TRAIL binding to its death receptors, DR4 and DR5 [132]. TRAIL in colorectal cancer, therefore, exemplifies a case of the same initiating signal inducing two forms of cell death, each having distinct characteristics and consequences. Much more work will be required before the implications are fully understood for colorectal and other cancers; however, it is tempting to speculate that these two cell death programmes may work in concert to promote oncogenic progression, especially given the fractional effects of TRAIL’s apoptosis-inducing properties even under saturating conditions. Thus TRAIL-induced apoptosis of a fraction of the cancer cell population may be complemented not only by consequent pro-malignant reparatory effects, as we have discussed, but also by further competition-based tumour evolution supported by TRAIL-induced entosis.

## 5. Conclusions and Future Perspectives

There is little doubt that much remains to be learned about the role of cell death processes in the pathophysiology of cancer. Similar to the two-faced ancient Roman god Janus, cell death appears to have a dual role in cancer biology with a complex picture emerging that takes us well beyond the simplicity of cancer-cell death being intuitively tumour suppressive. Many avenues require intensive investigation, not least the fundamental clarification of the molecular and cellular mechanisms underlying the control of irreversible cell deletion—as in the sculpting of developing organs—versus those governing the regenerative cell death that occurs when cells need to be replaced. Knowledge of the defining features of deletional, as compared to regenerative, cell death programmes may provide attractive targets for anti-cancer therapeutic strategies that promote the former and/or inhibit the latter. Despite a rich background in potential regenerative mechanisms involving responses of phagocytes and non-phagocytes in the ORN, definitive mechanisms are largely lacking. Key questions abound, including (but not limited to): (1) In different cancer types, to what extent and via what mechanisms is the TME’s response to apoptosis modulated at different stages of oncogenesis? (2) How does the TME’s response change in response to therapy-induced cell death? (3) What are the relative roles of different cell death and clearance processes during (a) primary and metastatic cancer growth and (b) cancer therapy? (4) To what extent is cell death molecular machinery deployed to aid cancer growth without driving cell death?

Important examples relevant to the last question are caspases, which are known to play apoptosis-independent roles, such as regulation of cell proliferation, cell fate determination and immune control [133]. Significantly, transformation following the phenomenon of ‘minority MOMP’ can occur, whereby some of the mitochondria of stressed cells become permeabilized, causing partial caspase activation that is insufficient to elicit cell death. Instead, this process can result in DNA damage, genomic instability and oncogenesis [72]. A further example, which we may regard in the present context as ‘apoptotic cell mimicry’ is the many-fold increased constitutive exposure of PS that has been reported on the surface of multiple types of healthy tumour cells and on tumour blood vessels [134,135,136,137,138,139,140]. The importance of this phenomenon has not been studied intensively but, as we have discussed, the potentially central role of PS and its receptors in the pro-oncogenic mechanisms of the ORN would suggest that mimicking apoptotic cells in this way could provide a further route to elicitation of tumour promoting responses without cell loss. Intriguingly, in an early study, tumourigenic human melanoma and carcinoma lines were not only estimated to display around 3–7 fold more PS in the external plasma membrane leaflet than a non-tumourigenic epidermal keratinocyte line, but also interacted with activated monocytes [134], a property reminiscent of apoptotic tumour cells. Furthermore, a combination chemotherapy approach using an anti-PS antibody has been shown to suppress prostate and hepatocellular cancer growth in mice in parallel with reversing TAM polarisation from pro- to anti-tumour (M2-like to M1-like) [141,142].

Future research into the yin and yang of cell death processes in cancer has many avenues to follow. The evolutionarily-conserved characteristics of apoptosis suggest that its functional properties in cancer have profound implications that are relevant to many, if not all, cancer types. It seems highly probable that continued effort to uncover the fundamental mechanisms of cell death-driven tumour-promoting, as well as tumour-suppressive, effects will not only improve knowledge of cancer biology but also identify better therapeutic targets that lead to novel combination therapies.

## Figures and Tables

**Table 1:** Examples illustrating the diversity of human cancers in which apoptosis in situ correlates with poor prognosis.

Cancer Type	References
Bladder carcinoma ^1^	[3,4]
Breast carcinoma ^1^	[5,6,7]
Colorectal carcinoma	[8,9,10,11,12]
Gastric carcinoma	[13]
Glioblastoma ^1^	[14,15,16,17]
Malignant mesothelioma	[18,19]
NHL ^1^	[20,21,22]
Non-small cell lung cancer	[23,24]
Pancreatic duct carcinoma	[25,26]
Squamous carcinoma of the tongue	[27]

^1^ Pro-oncogenic activity of apoptosis demonstrated in pre-clinical (including therapeutic) mouse models. NHL: non-Hodgkin lymphoma.

## Data Availability

Not applicable.
